# 

**Published:** 1880-05

**Authors:** 


### BOOKS AND PAMPHLETS RECEIVED.

A Manual of Pathological Histology. By V. Corine and L. Ranvier. Translated with notes and additions by E. D. Shakespeare, A.M. M.D. and J. Henry C. Simes, M.D. 360 illustrations on wood; price $6.50, pp. 784; sheep. Philadelphia: W. C. Lee. 1880.

Montreal General Hospital Reports, Clinical and Pathological. Edited by Wm. Osler, M.D. Vol. I. Montreal, Canada: Dawson Bros. 1880.

Annals of the Anatomical and Surgical Society. Vol. I. 1878–9. Brooklyn, N. Y. Published by the Society, 28 Madison street. 1879.

Brain Work and Overwork. By H. C. Wood, M.D. Price 50c. American Health Primer Series. Philadelphia: Presley Blakeston, 1012 Walnut street. 1880.

St. Bartholomew's Hospital Reports. Edited by W. S. Church, M.D., and Alf. Willett, M.D. Vol. XV. London: Smith, Elder & Co. 1879.

A Manual of Surgery. By W. Fairlie Clarke, M.D. Third edition; 190 engravings, all wood. Price $2.00. New York: G. P. Putnam's Sons. 1880.

Physiological Laboratory, Harvard Medical School, Boston. Collected Papers. 1873, 1879. For private circulation.

Minor Gynæcological Appliances and Operations for the Use of Students. By J. Halliday Croom, F.C.S.E.. Edinburgh: E. & S. Livingstone, 57 South Bridge. 1879. Price $1.75.

Sore Throat, its Nature, Varieties and Treatment. By Prosser James, M.D. Fourth edition, illustrated, hand colored plates. Philadelphia: Lindsay & Blakiston. 1880.

A Treatise on the Science and Practice of Midwifery. By W. S. Playfair, M.D., F.R.C.P. With notes and additions by Robert R. Harris, M.D. 185 Illustrations. Philadelphia: H. C. Lea. 1880.

A Treatise on Urinary and Renal Diseases, Including Urinary Deposits. By Wm. Roberts, M.D. Third American edition; price $4.00; cloth. Philadelphia: H. C. Lea. 1879.

Skin Diseases, a Manual for Students and Practitioners. By Malcolm Morris. Illustrated, price $1.75; pp. 320. Philadelphia: H. C. Lea. 1880.

Headaches, Their Natures, Causes and Treatment. By Wm. Henry Day, M. D. Third edition, illustrated. Philadelphia: Lindsay & Blakiston. 1880.

A Manual of Auscultation and Percussion. By Austin Flint, M.D. Second edition, revised. Philadelphia: H. C. Lea. 1880.

Observations on Fatty Heart. By Henry Kennedy, A.B., M.B. Dublin: Faunia & Co., 41 Grafton street. 1880.

A Practical Treatise on Nervous Exhaustion (Neurasthenia.) By Geo. M. Beard, A.M., M.D. New York: Wm. Wood & Co. 1880. Price $1.75.

The Microscope and Microscopical Technology. By Heinrich Frey. Translated and edited by Geo. R. Cutter, M.D. Illustrated, 183 wood engravings. New York: Wm. Wood & Co. 1880. Price $6.00.

The Student's Guide to Diseases of the Eye. By Edw. Nettleship, F.R.C.S. Eighty-nine illustrations. Philadelphia: H. C. Lea. 1880.

The Essentials of Anatomy. By Wm. Darling, M.D., F.R.C.S., and Ambrose L. Ranney, A.M., M.D. New York: G. P. Putnam's Sons. 1880.

A Practical Handbook of Medical Chemistry. By Wm. H. Greene, M.D. Philadelphia: H. C. Lea's Son & Co. 1880.

Clinical Lectures on Diseases of Women. Delivered in St. Bartholomew's Hospital. By J. Mathews Duncan, M.D., LL.D. Philadelphia: W. C. Lea 1880.

Dr. Denison's Climatic Map of the Eastern Slope of the Rocky Mountains in Wyoming, Colorado and New Mexico. Denver, Colorado. Price 50c.

Transactions of the Society of the Alumni of the Medical College of Ohio. Cincinnati, Ohio: *Cincinnati Lancet* Press Print. 1880.

Lectures on the Diseases of the Nervous System. By J. M. Charcot. Edited and translated by Geo. Sigerson, M.D. Illustrated. Philadelphia: H. C. Lea. 1880.

Atlas of Skin Diseases. By Louis A. Duhring, M.D. Part VI. Philadelphia: J. B. Lippincott & Co. 1879.

Atlas of Histology. By E. Klein, M.D., F.R.S., and E. Noble Smith, L.R.C.P., M.R.C.S. Parts VIII. and IX. Philadelphia: J. B. Lippincott & Co. 1879.

Atlas of Human Anatomy. Containing 180 Large Plates. Arranged according to Drs. Oesterreicher and Erdl. Explanatory Texts by J. A. Jeancon, M.D. Parts 1, 2, 3 and 4; 75 cents a part. A. E. Wilde & Co. Publishers, Cincinnati, Ohio.

A New Method of Permanently Removing Superfluous Hairs. By L. Duncan Bulkley, A.M., M.D. G. P. Putnam's Sons, New York. 1878.

On the Nomenclature and Classification of the Diseases of the Skin. By L. D. Bulkley, M.D.

Report of the Resident Physician of Brigham Hall, Canandaigua, N. Y.; 1880.

Sixth Annual Report of the Superintendent of the Cincinnati Sanitarium; 1879.

Report of the Condition of the Water in the First Ward of the City of Racine, Wis. By J. G. Meachem, JR., M.D.

### ANNOUNCEMENTS FOR THE MONTH.

### SOCIETY MEETINGS.

Chicago Medical Society—Mondays, May 3 and 17.

West Chicago Medical Society—Mondays, May 10 and 24.

### CLINICS.

MONDAY.

Eye and Ear Infirmary—2 p. m., Ophthalmological, by Prof. Holmes; 3 p. m., Otological, by Prof. Jones.

Mercy Hospital—2 p. m., Surgical, by Prof. Andrews.

Rush Medical College—2 p. m., Dermatological and Venereal, by Prof. Hyde; 3 p. m., Medical, by Dr. Bridge.

Woman's Medical College—2 p. m., Dermatological and Venereal, by Prof. Maynard; 3 p. m., Diseases of the Chest, Prof. Ingals.

TUESDAY.

Cook County Hospital—2 to 4 p. m., Medical and Surgical Clinics.

Mercy Hospital—2 p. m., Medical, by Prof. Quine.

WEDNESDAY.

Chicago Medical College—2 p. m., Eye and Ear, by Prof. Jones.

Rush Medical College—3:30 to 4:30 p. m., Diseases of the Chest, by Dr. E. Fletcher Ingals.

THURSDAY.

Chicago Medical College—2 p. m., Gynæcological, by Prof. Jenks.

Rush Medical College—3 p. m., Diseases of the Nervous System, by Prof. Lyman.

Eye and Ear Infirmary—2 p. m., Ophthalmological, by Dr. Hotz.

Woman's Medical College—3 p. m., Surgical, by Prof. Owens.

FRIDAY.

Cook County Hospital—2 to 4 p. m., Medical and Surgical Clinics.

Mercy Hospital—2 p. m., Medical, by Prof. Davis.

SATURDAY.

Rush Medical College—2 p. m., Surgical, by Prof. Gunn.

Chicago Medical College—2 p. m., Surgical, by Prof. Isham; 3 p. m., Neurological, by Prof. Jewell.

Woman's Medical College—11 a. m., Ophthalmological, by Prof. Montgomery; 2 p. m., Gynæcological, by Prof. Fitch.

Daily Clinics, from 2 to 4 p. m., at the Central Free Dispensary, and at the South Side Dispensary.

